# Attentional switching in larval zebrafish

**DOI:** 10.1126/sciadv.ads4994

**Published:** 2025-10-03

**Authors:** Kumaresh Krishnan, Akila Muthukumar, Scott Sterrett, Paula Pflitsch, Adrienne L. Fairhall, Mark Fishman, Armin Bahl, Hanna Zwaka, Florian Engert

**Affiliations:** ^1^ Molecular and Cellular Biology Dept., Harvard University, Cambridge, MA 02138 USA.; ^2^ Stem Cell and Regenerative Biology Dept., Harvard University, Cambridge, MA 02138 USA.; ^3^ Dept. of Neurobiology and Biophysics and Computational Neuroscience Center, University of Washington, Seattle, WA 98195 USA.; ^4^ Leibniz Instit. for Neurobiology, Magdeburg 39118, Germany.; ^5^ Neurobiology, Universitat Konstanz, Konstanz 78464, Germany.

## Abstract

Decision-making strategies in the face of conflicting or uncertain sensory input have been successfully described in many species. We analyze large behavioral datasets of larval zebrafish engaged in a “coherent dot” optomotor assay and find that animal performance is bimodal. Performance can be separated into two “states”—an engaged (attentive) state with high performance, where fish consistently turn in the direction of coherent motion, and a second, disengaged (inattentive) state, where performance drops to chance. A hidden Markov model is sufficient to model these transitions and can be incorporated into a drift-diffusion model framework that has previously been used to model perceptual decision-making in larval zebrafish. Furthermore, we fit a mixture model of performance distributions and extract two latent variables termed “focus” and “competence” that are largely influenced by parents and environmental context, respectively. This quantitative framework can potentially help to identify a genetic basis and a neural mechanism for attention that extends across organisms.

## Teaser

Attentional switching in larval zebrafish suggests a basis for focus and competence while performing a task.

## INTRODUCTION

All organisms receive sensory inputs that are processed to produce behavioral responses in the form of motor movements. Such motor outputs often result as a consequence of spatial and/or temporal integration of noisy sensory information. Algorithms underlying sensory integration and perceptual decision-making have been studied in a variety of organisms such as rodents (*1*–*4*), flies (*5*–*7*), and primates (*8*–*10*), as well as in the larval zebrafish (*Danio rerio*) (*11*, *12*). In zebrafish, such decision-making can be readily studied in the context of the coherent-dot optomotor response (OMR), where zebrafish follow the direction of a global motion cue, presumably to stabilize their position in a moving current. This robust and reliable innate behavior provides an ideal framework to study the temporal integration of noisy evidence in the context of decision-making. Given that larval zebrafish swim in discrete events, termed bouts, each of these bouts can be considered a readout of an underlying decision-making process, thereby greatly facilitating the collection of large numbers of discrete choices within a limited time frame.

There is evidence to suggest that organisms may be attentive toward specific information in certain periods and ignore the same information in other periods (*13*, *14*). Such attention-based frameworks have been developed for decision-making in rodents, primates, and humans as well (*15*–*17*). Here, we adopt a framework that models the transition between attentive and inattentive states using a simple two-state hidden Markov model (HMM). We find that this approach succeeds in capturing the average trends in performance, as well as the variance of observed individual swim decisions throughout the experiment. This framework implies that errors can arise either from noise in the sensorimotor transformation or from a transition into inattentiveness where the stimulus is ignored entirely.

We find that such stochastic state transitions can be readily combined with an existing drift-diffusion model (DDM) framework that has previously been used to model perceptual decision-making in larval zebrafish (*11*). Similarly, approaches in other organisms have treated such decision-making in the context of DDMs as a single strategy applied repeatedly to describe the processing of sensory information (*18*–*22*). We find that the addition of the DDM succeeds in replicating some aspects of the low-level structure and variance of observed individual swim decisions that a simple HMM alone is not designed to capture.

Last, we present evidence supporting the hypothesis that the degree of inattentiveness is linked to parental contributions, while competence is linked to environmental context. Here, we define “competence” as quantifying the animal’s intrinsic ability to make use of sensory information. By investigating such questions in larval zebrafish, we can leverage access to a tractable system for brain imaging studies that will allow us to eventually uncover circuits and mechanisms for decision-making and attention, which will go well beyond the promising but preliminary insights obtained in a previous study (*11*).

## RESULTS

### The performance of larval zebrafish in the OMR is suboptimal

**Fig. 1.**
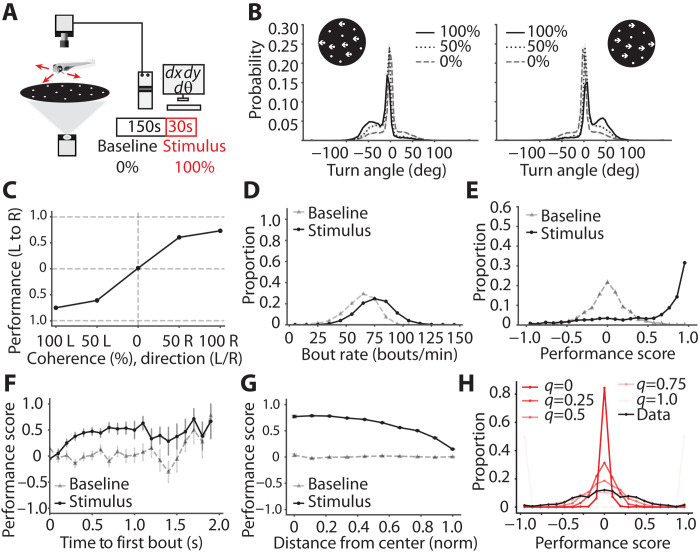
Characterizing OMR decision-making. (**A**) Schematic of the setup used to record free swimming behavior from larval zebrafish in response to dot motion stimulus. (**B**) Distribution of angle turned in each bout for leftward and rightward moving dots—the dashed line is 0% coherence, the dotted line is 50% coherence, and the solid line is 100% coherence. (**C**) Psychometric curve for average performance at each coherence level—for visualization purposes, only the 0 (chance)–to—1.0 (fully correct) region of scores are shown on the *y* axis. (**D**) Distribution of bout rates observed across all fish during free swimming—gray triangles show the baseline bout rate, and black circles show the stimulus bout rate for 100% coherence. (**E**) Distribution of performance scores—gray triangles show the 120-s baseline period, and black circles show the 30-s stimulus period of 100% coherence. (**F**) Variation of performance with time taken for the first bout after stimulus onset—gray triangles show the baseline period, and black circles show the stimulus period of 100% coherence, averaged in 100-ms bins. (**G**) Variation of performance with distance of fish to the center of the dish—gray triangles show the baseline period, and black circles show the stimulus period of 100% coherence. Pooled data across *n* = 64 fish and 30 trials for all plots; errors are SEM over trials in each bin for [(F) and (G)]. (**H**) Comparison of theoretical binomial processes for different probabilities to switch between fully left and fully right biased states. Black circles show experimental data. The distribution width increases with *q*—the probability of turning in a given direction (*q* is not direction specific as we look at the performance score).

To quantitatively evaluate the performance of larval zebrafish in an optomotor context, we leverage the random dot motion assay that allows one to calibrate the difficulty of the stimulus used to evoke the OMR. Specifically, we present whole-field dot motion at different coherence levels at the bottom of a circular dish and record stimulus-induced turning behavior. Turns are easily translated into a performance score depending on whether individual turns align with, or go against, the stimulus direction (Fig. 1A). A single trial in the experiment consists of 150 s in which dots move with 0% coherence (referred to as the baseline period), followed by 30 s in which dots move with constant nonzero coherence (referred to as the stimulus period), either to the left or to the right with respect to the zebrafish’s body. The fish’s position and orientation are both tracked online, which allows us to implement a closed-loop configuration: After each turn, the motion direction of the dots is updated to remain orthogonal to the fish’s body axis such that the motion is always perpendicular to the orientation of the animal. We show that this stimulus evokes continuous turning behavior while the stimulus is present and that the proportion of turns in the direction of the stimulus increases with the coherence of dot motion (Fig. 1B). If motion coherence is reduced from 100 to 50%, the fraction of correct turns drops accordingly, and at 0% coherence, turn probabilities approach chance levels, as evidenced by the symmetry of the turn angle distribution around zero (fig. S1A). Performance in the OMR is calculated as the fraction of turns in the direction of the stimulus (rightward, greater than 0; leftward, lesser than 0; equal contribution, 0). This is rescaled to a score spanning −1.0 to 1.0 (i.e., from fully against the stimulus direction to fully aligned with the stimulus direction). The psychometric curve (Fig. 1C) shows that the performance in the optomotor task is not perfect even at 100% coherence, regardless of the stimulus direction. All further analysis and modeling are carried out on data collected in response to a fully coherent motion stimulus, i.e., coherence of 100%, with the exception of the psychometric curve.

A single swim in larval zebrafish is a discrete movement termed a “bout,” which corresponds to a burst of tail movement resulting in either a turn or forward movement. The terms “swim” and “bout” are used interchangeably while referring to these discrete events. We observe that zebrafish bouts occur at a higher rate during the stimulus period than in the baseline period (Fig. 1D). Mean bout rates are 62.90 +/− 14.50 bouts/min during the baseline period and 75.22 +/− 16.35 bouts/min during the stimulus. By assigning a random target direction for the baseline period, we show that performance scores are normally distributed around the chance level (zero). During the stimulus period, when fish are exposed to directed dot motion at 100% coherence, the performance scores are strongly skewed toward higher values (Fig. 1E). Nonetheless, we find that 7% of scores are still below −0.5 (Fig. 1E), which means that fish, even in the presence of a 100% coherent stimulus, make many “wrong” decisions.

To test the previously shown role of temporal integration in decision-making (*11*, *12*), we measure performance as a function of the waiting time until the initiation of the bout (Fig. 1F). In agreement with earlier results (*11*, *12*), we find that the performance during the stimulus period improves as the waiting time increases, and performance saturates beyond 0.75 s after stimulus onset, with increased noise beyond a waiting time of 1 s as the number of bouts with this waiting time is small (fig. S1B). We observe that this improvement in performance is true only for the first bout after stimulus onset and not for subsequent ones (fig. S1C). This reemphasizes that zebrafish accumulate evidence for motion until a certain threshold, which translates to performance improvement until a certain time after stimulus onset. As expected, no such trend is present in the baseline period where there is no consistent target direction.

Furthermore, we analyze fish swimming behavior in relation to the distance to the wall, because it might serve as a potential distractor and interfere with the assay. We find that as fish get closer to the wall, they tend to bias their turns away from this physical obstacle (*23*). Consequently, performance in the optomotor task approaches chance levels at the point of contact (Fig. 1G).

We next test whether left-right choices during baseline swimming can be explained by a coin flip (or random) model. We find that the distribution of choices is narrower in the coin flip model than the experiment, where we match the number of bouts observed in the experiment to appropriately select the number of coin flips (Fig. 1H). This aligns with prior research in the field indicating that zebrafish swimming has a memory component, i.e., fish display an increased probability to swim in the same direction over consecutive swims (*24*). This behavior can be captured by a process where each fish displays a bias for the same direction over consecutive turns and where this bias can switch to the opposite direction after an extended time period (*24*). Including this memory component leads to a sequence of turns in the same direction and widens the distribution of baseline performance scores, as values closer to −1.0 or 1.0 become more likely (fig. S1, C to E).

### Performance score distributions in the optomotor task indicate attentional switching

**Fig. 2.**
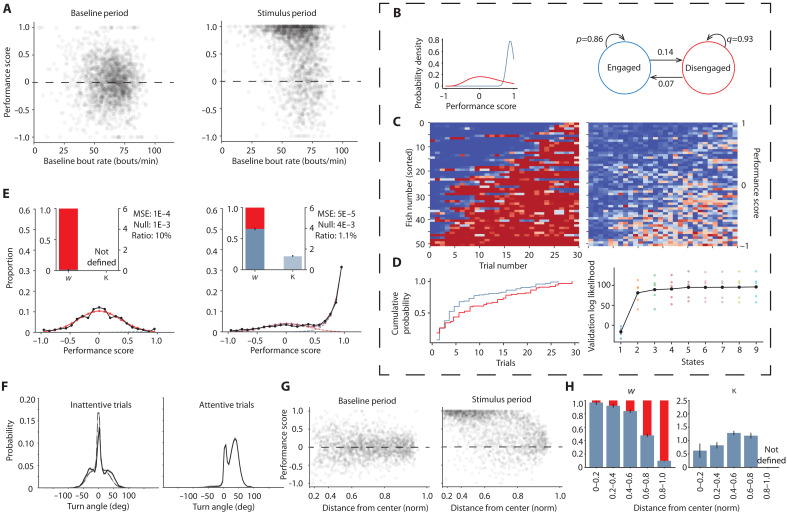
Attentional switching in the OMR. (**A**) Point cloud of all 1920 trials showing the distribution of performance scores against bout rate in the baseline period for (left) the performance evaluated against a random target in the baseline period and (right) the performance evaluated in the stimulus period. (**B**) (Left) Gamma distributions inferred from the HMM that best fits the experimental data and (right) compact description of the proposed attentional switching framework. (**C**) (Left) Posterior probabilities for engaged (blue) and disengaged (red) states based on the HMM (right) performance score across trials for each fish sorted by average score. (**D**) (Left) Dwell time distribution for the engaged and disengaged states showing the probability of staying in a state for a certain trial length and (right) log likelihood of the HMM with varying number of states. (**E**) Distribution of performance scores corresponding to (left) the baseline period and (right) stimulus period shown in black circles along with fit lines for the Gaussian-gamma mixture model with the Gaussian component in red and the gamma component in blue. The inset shows the value of *w* in blue indicating the weight of the Gaussian and κ indicating the shape parameter of gamma distribution. The MSE for fits and comparison to a null model (see Materials and Methods) is also reported. (**F**) Turn angle distributions for all bouts occurring in trials classified as (left) inattentive in solid and baseline in dashed (right) attentive. (**G**) Point cloud of the average radius of bouts in a trial against the resulting average performance during (left) the baseline period and (right) stimulus period. (**H**) Performance score distributions within each average radius subset of 0, 0.2, 0.4, 0.6, 0.8, and 1.0.

Might the baseline behavior provide a predictive estimate of the animal’s internal state during the subsequent task? We estimate the “baseline state” by computing the swim rate during the baseline period (baseline bout rate). We then plot the baseline bout rate for each trial against the performance score in the subsequent stimulus period as a “point cloud” that included all 1920 trials (Fig. 2A). Several features become apparent.

First, during the baseline period, the bout rate is normally distributed around 60 bout/min. The chance level (score of zero) performance scores during this period are captured, as expected, by a Gaussian distribution centered at zero (Fig. 2A, left).

Second, during the stimulus period (Fig. 2A, right), the performance score distribution can be visually separated into two clusters, one that mirrors the normally distributed cluster at chance, which we call the “disengaged” cluster, and a separate, high-performance cluster, with performance score values much closer to 1. We refer to this as the “engaged” cluster. These two separate clusters suggest that the suboptimal performance of fish in our assay can be explained by the animals not attending to the task at all during a subset of time intervals.

Third, the baseline bout rate in the “disengaged” cluster is shifted to higher values when compared to the baseline cluster (Fig. 2A, compare left to right; see also Fig. 1D), indicating that the state of these animals is not identical to the baseline condition, which allows us to infer internal motivational states on the basis of baseline swimming rates alone.

Last, the performance values in the “engaged” cluster show a negative correlation with baseline bout rate, indicating that slow swim rates during the baseline period predict higher performance during the stimulus (fig. S2K, top).

To quantitatively model the transition between the “engaged” and “disengaged” clusters, we use a two-state HMM with gamma emissions, which captures the structure in the stimulus trials (Fig. 2B, left). While the disengaged cluster is well modeled by a Gaussian distribution, a gamma function was chosen as it can both be approximately Gaussian and model the asymmetric, high-performance “engaged” cluster. This binomial process can be represented by a two-state transition model with transition rates *p* and *q* (Fig. 2B, right). The HMM-based posterior probabilities for the two states (Fig. 2C, left) show a qualitative correlation with the performance score sequences obtained from the experiment (Fig. 2C, right). Furthermore, there is comparable persistence in both states as shown in the dwell time distributions (Fig. 2D, left). There are no engaged state assignments longer than 26 trials, which is reflected in the dwell time distributions. Around 60% of the engaged trials last for less than 10 trials (Fig. 2D, left). We further show that including additional states does not provide added benefits (Fig 2D, right). This suggests that our two-state model of behavior is the most concise description of this state-dependent performance phenomenon.

To extract the parameters of the underlying Gaussian and gamma distributions, we fit a Gaussian-gamma mixture model to the performance score distribution across all 1920 trials for baseline (Fig. 2E, left) and stimulus periods (Fig. 2E, right). As expected, two aspects in larval zebrafish performance are captured—a smaller fraction of scores centered around the chance level and a larger fraction of scores skewed toward higher performance values (Fig. 2E, right).

This fit then allows us to characterize the score distributions by two parameters, *w* and κ. In simple terms, *w* indicates the weight of the gamma distribution relative to the Gaussian distribution and can be interpreted as a measure of the level of engagement (“focus”). κ indicates the extent to which performance scores are skewed toward higher values in the gamma distribution—a measure of performance when engaged (“competence”) (Fig. 2E, inset left, right). We propose that “focus” and “competence” serve as independent descriptors of performance in a given task that can also be applied to generic tasks in higher organisms. Notably, this approach allows us to rigorously quantify the state of the animal’s engagement, which permits a statistical comparison across different experimental contexts and across different fish strains.

We also find that the turn angle distribution for bouts in the disengaged cluster (Fig. 2F, left) is notably similar to the baseline turn distribution shown in Fig. 1B. The attentive trials show almost no turns against the stimulus direction and reflect the excellent performance score when engaged (Fig. 2F, right).

We next attempted to extract and identify state transitions within a trial. However, the fact that the turn angle distribution has a large peak at zero in both attentive and inattentive trials makes it difficult to classify an individual bout as belonging to one of these states purely based on the magnitude of the angle turned. This overlap makes it difficult to examine attentional switching within a trial. Therefore, we limit our analysis to attentional switching between trials.

To test the sufficiency of the two-state attentional switching model to capture a lower-level structure in our data, such as the drop in performance as a function of baseline bout rate, we implement a simple generative process using the HMM with gamma emissions (see Materials and Methods). The results indicate that the lower-level structure within the high-performance cloud is not captured by this approach (fig. S2A) and requires a more detailed description. To accomplish this, we leverage a prior modeling framework, the DDM, which can be easily combined with our state transition model (see Supplementary Text 1.1 to 1.4) to capture the lower-level structure (fig. S2, B, C, and H). To do so, the attentive state follows the typical DDM motion integration dynamics, while in the inattentive state, the visual information containing the motion signal is gated. The transition between the two states is now governed by rates *p* and *q* that encapsulate the baseline bout rate and time spent in the experiment.

On the basis of our initial observation that performance drops with distance from the center of the dish, we analyze how performance varies with distance from the wall in each trial. During the baseline period, the performance scores are symmetrically spread around the chance level, as expected, regardless of the distance from the wall (Fig. 2G, left; center, 0; wall, 1.0). During the stimulus period, we notice a clear clustering of higher performance at the center of the dish (distance << 1) and chance-level performance in the vicinity of the wall (Fig. 2G, right). We believe that this indicates that zebrafish can get distracted by the wall, leading to a drop in performance. The normally distributed shape of the low-performance cloud close to the wall suggests that this drop in performance is attributed to a loss of “focus” (decrease in *w*) and not a reduction in “competence” (decrease in κ). We consider subsets of these data lying within a specific radius (0 to 0.2, 0.2 to 0.4, 0.4 to 0.6, 0.6 to 0.8, and 0.8 to 1.0) and compute the performance score distributions for each subset (Fig. 2H). This is compatible with our hypothesis that visual distractors from the wall can serve to disengage the fish from the task and provides further evidence that the performance decrease can be attributed to a greater loss in focus, perhaps caused by a change in behavioral priorities.

### Inheritance influences the level of “focus,” while the environment influences the level of “competence”

To distinguish the role of inheritance versus environment with the help of our assay, we compare two zebrafish populations, both of the same wild-type (WT) strain but bred independently in separate facilities for many generations, resulting in different genetic backgrounds. Each of these two populations is housed in different locations and environments, named “A” and “B” for simplicity.

**Fig. 3.**
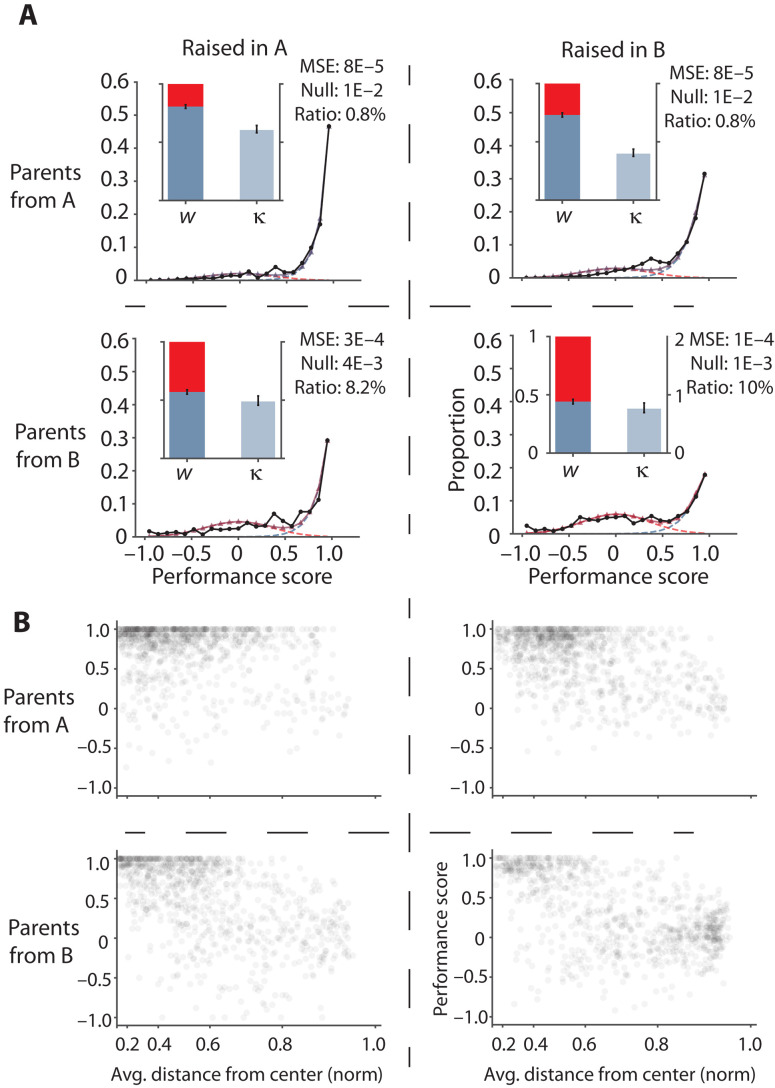
Effect of inheritance versus environment. (**A**) Performance score distribution and value of *w* and κ compared for parents from A and fish raised in A (top left), parents from A and fish raised in B (top right), parents from B and fish raised in A (bottom left), and parents from B and fish raised in B (bottom right). (**B**) Performance variation against distance from the center of the dish for every (fish and trial) pair in the experiment for the same raising conditions as the above panel. *n* = 32 fish and 30 trials. Error bars are SEM for parameter estimates over 5000 fits on bootstrapped data. The MSE is for fit versus experimental data. Null is the MSE of a null model (see Materials and Methods) versus experimental data. Statistical tests across groups are permutation tests (see Materials and Methods).

Through our optomotor assay, we find that animals collected from “A” display significantly higher values for *w* and κ, indicating that they are superior in both “focus” and “competence” when compared to fish collected from “B” (compare Fig. 3A, top left and bottom right). Next, we collected fertilized fish embryos from each location, split them into two groups, and raised one of the groups in their home facility as a control and the other in the opposite location to test for putative effects of a change in environment.

Through this preliminary study, we find that “focus,” *w*, reflects the location of origin of the parents but not the raising environment. Thus, *w* is large for the progeny of parents from location A and small for those with parents from location B and that distinction is retained even for the progeny raised in the alternate location. Competence κ, on the other hand, depends strongly on the environment (raising conditions and environmental impact) and shows much smaller dependency on parental strains (Fig. 3A). The transfer of fish from A to B induces a significant decrease in κ, whereas moving embryos from B to A results in an increase in κ, signifying an improvement in “competence.” The statistical significance of the differences in *w* and κ across the groups is shown in Table 1 (also see Materials and Methods). These tests show that the facility of parental origin may influence *w* to a larger extent, whereas the environment of raising and testing primarily influences κ.

**Table 1.** Statistical significance for κ and *w* values across groups from Fig. 3. Testing all groups from swapped raising for statistical significance in *w* and κ. AA means parents from A and raised in A, AB means parents from A raised in B, and so on; all tests are permutation tests (see Materials and Methods). We observe that weights for fish with parents from B are much lower than weights for fish with parents from A. Bold values are *P* values < 0.05.

Comparison	*P*_bootstrap_ value for parameter κ	*P*_bootstrap_ value for parameter *w*
AA vs BB	**0.023**	**<1/10,000**
AA vs AB	**0.021**	0.254
AA vs BA	0.345	**0.001**
BB vs BA	**0.138**	**0.046**
BB vs AB	0.718	**<1/10,000**
AB vs BA	0.175	**0.018**

We therefore hypothesize that the ability to stay “focused” is largely inherited from the parents, whereas “competence” when engaged in a specific task depends largely on the environmental upbringing. In this context, the small but significant change in *w* that occurs when animals are raised in a different environment (particularly for fish from facility B, see Fig. 3A) either can be caused by residual effects of the extensive difference in sensory stimulation experienced in the new environment or, alternatively, could be caused by different environmental conditions, such as pollutants, salinity, or temperature, during early embryonic development, thereby inducing epigenetic changes. To our knowledge, food is the only well-controlled variable between the two facilities, where fish from facility A were fed paramecia and fish from facility B were fed rotifers. Together, these results suggest that “focus” and “competence’ may be controlled separately by “Nature” and “Nurture,” respectively.

Separately, our finding that the wall can serve as a source of distraction leading to poor performance, caused by the loss of “focus,” is also confirmed in these experiments (Fig. 3B), where the density of the high-performance cluster close to the “ceiling” (performance, 1) is indicative of high κ, and the density of the low-performance cluster is indicative of a low *w* close to zero. To probe the influence of inheritance and acute sensory perturbations on *w* and κ, we next test a range of experimental and genetic perturbations and ask how “focus” and “competence” are specifically affected by such manipulations.

### Screening for performance and attention levels

In attempting to isolate differences between groups, we anticipate that the large and ubiquitous variation in animal performance, which complicates rigorous ethological studies in all species, will manifest also in our experiments through variations of both *w* (“focus”) and κ (“competence”).

**Fig. 4.**
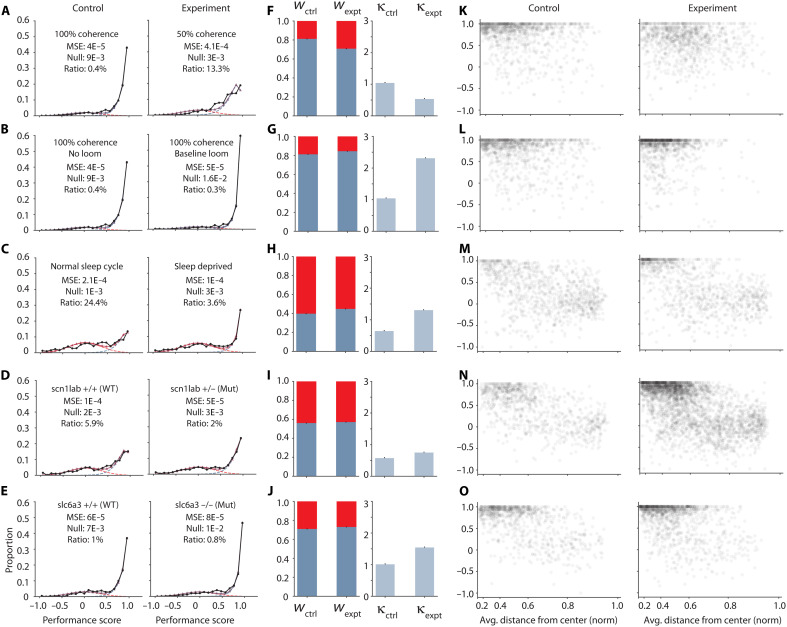
Effect of acute and genetic perturbations. (**A** to **E**) Fitted performance distributions for all experimental manipulations—black circles are data, colored triangles are fits, and dashed lines are fit components. Left: control; right: experiment. (A) Control: 100% coherence, *n* = 32 fish; experiment: 50% coherence, *n* = 32 fish. (B) Control: 100% coherence, *n* = 32 fish; experiment: looming distraction, *n* = 32 fish. (C) Control: 100% coherence and normal sleep cycle, *n* = 32 fish; experiment: 100% coherence and sleep deprivation, *n* = 32. (D) Control: 100% coherence and *scn1lab*^+/+^, i.e., WT, *n* = 29 fish; experiment: 100% coherence and *scn1lab*^+/−^, i.e., mutants, *n* = 99 fish. (E) Control: 100% coherence and *slc6a3*^+/+^, i.e., WT, *n* = 28 fish; experiment: 100% coherence and *slc6a3*^−/−^, i.e., mutants, *n* = 39 fish. (**F** to **J**) Comparing fit parameters *w* between control and experiment—error bars are SEM for parameter estimates over 5000 fits on bootstrapped data. Thirty trials for all experiments. (**K** to **O**) Comparing performance variation with the distance to the wall for control and experimental groups. The MSE is for fit versus experimental data. Null is the MSE of a null model (see Materials and Methods) versus experimental data. Statistical tests across groups are permutation tests (see Materials and Methods).

This general problem can be largely addressed by systematically comparing the test group to a sibling control cohort that shares most parental and environmental contributions. We take advantage of the large clutch size of zebrafish (~50 to 100 offspring per mated pair), dividing each clutch into two groups where one serves as a control and the other is tested for behavioral effects of a specific perturbation. Performance values in the sibling control cohorts, as shown in the left column of Fig. 4, show a wide distribution of performance metrics, which emphasizes the importance of comparing experimental results against sibling controls. We further leverage the independence of *w* and κ values, which allows us to draw conclusions as to whether the applied perturbations affect “focus,” “competence,” or both. See Materials and Methods for statistical analysis details.

As a first control experiment, we reduced coherence levels of the stimulus from 100 to 50% and observed that the drop in performance was explained mostly by a decrease in κ (*P* < 1 × 10^−4^). *w*, which was already very large in the control group for this particular clutch of animals, also decreased but was not statistically significant (*P* = 0.061; Fig. 4, A and F). This is consistent with the idea that the performance drop can be attributed to an increased difficulty in the stimulus. The lack of a pronounced wall effect in this perturbation is consistent with high *w* values (fish are not distracted).

The drop in κ, on the other hand, results in an increased vertical spread of the high-performance cloud (Fig. 4K). The performance scores observed in Fig. 4A strongly argue against the possibility that the drop in performance seen close to the wall could be explained by a reduction in overall motion information, because the fish’s visual field is reduced to half when swimming next to the wall: The performance drops to chance levels near the walls. However, a 50% drop in input strength (or coherence) has a much more moderate effect as seen in Fig. 4A.

To test how explicit distractors during the baseline period may affect performance during the subsequent stimulus period, we expose the fish to a series of looming stimuli during the baseline. These stimuli consist of dark disks that approach the fish with increasing size, mimicking predator approach and triggering escape swims away from the threatening objects. When we measure performance during the stimulus period, where no looming stimuli were shown, we find a marked increase in κ (competence) (*P* = 0.001; Fig. 4B), leading to a significant improvement in net performance, with no significant change in *w* (focus) (*P* = 0.528). Consistent with these observations, zebrafish also display a greater density of scores in the high-performance region (Fig. 4L). Notably, for the looming distraction, we use the same sibling controls as in the reduced coherence task and only split the test groups into two different cohorts.

Next, we compared sleep-deprived fish (constant light during the night) to a control group that underwent a regular sleep cycle (9 a.m. to 11 p.m. light). We find that sleep-deprived fish show significantly improved performance values (*25*), which are mostly attributed to an increase in κ (*P* < 1 × 10^−4^; Fig. 4, C and H), whereas sleep deprivation seems to have no significant effect on *w* (*P* = 0.528), which was particularly low for this specific clutch also in sibling controls. Again, the large increase in κ is reflected by a proportional increase in density of the high-performance cloud, whereas distractions by the wall are similar in perturbed and unperturbed animals.

Last, we test two mutant lines, *scn1lab* and *slc6a3*, affecting sodium channel units and dopamine transporters, respectively (*26*–*29*). These mutations have been implicated in autism and schizophrenia, and they provide a useful test case for our analysis framework. Notably, we find that *scn1lab* mutant animals, when compared to WT siblings, show a moderate increase in κ (*P* = 0.002) and no significant change in *w* (*P* = 0.831), leading to a net increase in performance (Fig. 4D). The higher density of all clouds describing mutant animals is explained by the heterogeneous number of available fish in each genotype (29 WT and 99 heterozygous). *slc6a3* mutants show an even stronger improvement in κ value (*P* = 0.021) compared to WT siblings, and also here, *w* appears to be unchanged (*P* = 0.747; Fig. 4, E, J, and O). These mutations are also known to affect the circuitry of the developing brain (*26*–*28*).

None of our explicit perturbations significantly affected *w* values, and changes in performance could all be explained by related changes in κ. However, *w* values showed considerable variation in control groups across all experimental paradigms, where individual clutches were always collected from different parents. This supports the notion that inheritance appears to dominate the innate ability to “focus” (i.e., the *w* values outlined in Fig. 4), whereas the ability to perform while engaged is more plastic and heavily influenced by acute environmental perturbations.

Furthermore, it is interesting that all perturbations tested led to a significant increase in net performance, with the expected exception where motion evidence was dropped from 100 to 50% coherence. While these results will require more rigorous testing, this method of screening can provide reasonable insight into changes in “focus” and “competence” during decision-making tasks and inform subsequent investigations.

In summary, we propose that attentional switching is an important component of decision-making strategies in larval zebrafish. Our ability to parse behavioral performance into two independent key parameters *w* and κ that correlate with the level of “focus” and “competence,” respectively, is critical to quantitatively capture this behavior. Last, we find that the wall provides a natural source of distraction that can be leveraged to facilitate and enhance our ability to extract and validate these parameters.

Together, we can use this approach as an effective screening pipeline for experimental and genetic perturbations. We emphasize that mistakes need not arise from the inability to perform a task but may simply occur because of the lack of attention—or paying attention to a more relevant stimulus.

## DISCUSSION

Here, we use larval zebrafish to delineate two components of performance—focus and competence—in a sensory decision-making task that is well described in primates and rodents. Specifically, we show that genetic and environmental factors influence focus and competence, respectively. The analysis relies on a modeling framework that captures the level of engagement or disengagement in the task (OMR in our case). This approach shows that the suboptimal performance can be explained largely by fish transitioning into a state of disengagement where they appear to ignore the stimulus and their performance drops to chance levels.

Other approaches have been proposed to explain changes in performance scores in decision-making tasks. For example, the drop in performance can be explained by fluctuations exclusively in the current sensory input (*30*) or, alternatively, by modulating the evidence accumulator on the basis of past trial outcomes (*4*, *21*, *31*). However, the drop in performance to chance levels observed in our experiments suggests that the animals are not merely compromised in their ability to solve the task but that they disengage completely.

We note that the lack of focus and the lack of competence are two orthogonal reasons for failing in any sensory decision-making task and that any modeling framework needs to account for both. We believe that separating any performance evaluation (be it in humans, primates, rodents, or fish) into these components is useful to a large number of scientists who are interested in the neural processes of decision-making. Here, we explicitly test this approach by modeling the decision process itself through a DDM and using an HMM to simulate the state transition between engaged/disengaged states. We acknowledge that other modeling approaches can be selected for either of these processes where the HMM and DDM frameworks are particularly popular and successful implementations.

In our case, we have remained largely agnostic about the explicit factors that influence the transition probability between engaged and disengaged states in the HMM. However, we identified wall proximity, time in the tank, and baseline swim rates as contributing factors that were extracted from experimental data.

The phenomenon of an animal transitioning into a “disengaged” state was recently described in mice undergoing a two-choice visual decision-making task of a comparable nature. In this study, a generalized linear model-HMM framework (GLM-HMM) was used as an extended version of the classic “lapse” model (*15*). The GLM-HMM is compatible with our approach of treating inattention as a state transition from actively solving the task. As we demonstrate, our data do not justify a further split of the inattentive state into discrete strategies as in (*15*). Here, we have used the DDM rather than the statistical characterization of a GLM as this builds on previous work by specifying an explicit algorithm to solve the task while engaged. Our framework need not be restricted to two choice problems but can be generalized to any task where an algorithm to “solve” the task exists alongside a transition to inattentiveness.

Now, our model does not capture individual decisions but evaluates performance within a time window. This approach is data hungry—we currently gather performance scores from 1920 trials (time windows). Larval zebrafish facilitate these high-throughput experiments, but extending this framework to other organisms while maintaining the high throughput requires careful experimental design. While we find that modeling at the trial-level resolution is readily interpretable, assigning individual decisions to a single state can also be attempted through likelihood estimation methods.

### Circuit implementation

In terms of neural implementation, the DDM is rooted in a realistic circuit model (*11*), and the switch to inattentive states can be readily implemented into this framework. Visual information flows through four stages in this circuit model. First, whole-field motion is represented by specific neurons in the pretectum, likely relayed by whole-field motion–selective retinal ganglion cells (*32*). Second, this information is accumulated by a dedicated “integrator” circuit in the ventral hindbrain. Third, inhibitory dynamic threshold neurons are identified, whose activity is combined with the excitatory integrator neurons to target “motor output” neurons, which represent the fourth stage.

We propose that there exists a “gating network,” composed of modulatory neurons that have been found in the area postrema or nucleus coeruleus, both located in the medulla oblongata, that have already been shown to silence inputs into motor nuclei in the context of “giving up,” i.e., the induction of passivity by futile actions (*33*, *34*).

Such networks can exert their influence at any or all stages of the DDM circuit model. They either can suppress activity in the pretectum; can gate the input into the integrator, thereby suppressing evidence accumulation; could drive activity in the dynamic threshold neurons; or could directly synapse onto the motor output neurons and prevent the execution of directed turns.

This makes explicit and clear predictions about neural network changes in a larval zebrafish that is performing the OMR while brainwide activity patterns are being recorded (*11*, *12*, *32*, *35*). To execute these informative experiments, it is necessary to identify transitions between engaged and disengaged states in head-fixed larvae, a process that is tractable, given the findings presented here.

### Switching between states

We suggest that fish in the “inattentive” state are not generally disengaged with the world but rather direct their attention to a stimulus of a different modality or a different contextual value. Fish, like all other animals, need to solve more problems in life than simply holding position in a moving stream, and it is most likely an adaptive property of the animal if the sensory machinery can be retargeted to prioritizing a problem of a different nature. This can be contextualized within the exploit versus explore framework, which usually describes “value-based” switching from one task to another. For example, larval zebrafish need to avoid looming objects when predation is likely (*36*–*38*), they need to respond to the presence of conspecifics to avoid crowding or to facilitate schooling (*28*, *39*), and they need to pursue prey to eat (*40*, *41*). As such, we suggest that the “inattentive” state of our animals is best described by a transition into a state of different behavioral priority.

### Beyond zebrafish

Most, if not all, animals solve existential behavioral problems in series rather than in parallel. Fight or flight, freeze or escape, and pursue or abort are just a few of countless examples that illustrate this point. For many behaviors, it is quite difficult, if not physically impossible, to execute them in parallel, which is also reflected in the way many underlying neural circuits are implemented. Many examples of “winner takes all” circuits exist, where mutual long-range inhibition guarantees the temporary survival of only a single, dedicated subcircuit at any given time (*42*–*46*). Our example of a larval zebrafish transitioning into an “inattentive” state is likely just another case of a different subcircuit, distinct from the one dedicated to integrating noisy visual motion, taking over and dominating the sensory motor program. Such alternative inputs could originate from somatosensory regions involved in rheotaxis (*47*) or olfactory centers that guide navigation through salt gradients (*48*), among many others.

### Inheritance versus environment

We have collected consistent evidence that the fish’s likelihood of transitioning into an inattentive state appears to be primarily influenced by parental contributions, suggesting that the value for *w*, i.e., the ability to focus, is an inherited trait. These fish strains have been raised in different facilities for many generations, so they have different genetic backgrounds. The difference in environmental raising conditions may induce epigenetic changes in the parental germ cells or the developing zygote. A resolution of whether the heritable effects are due to genetic background or epigenetic influences would require careful control of the parental environment, followed by an analysis of potential resulting effects on focus in the offspring.

Both *w* (focus) and κ (competence) reflect the underlying neuronal circuitry. Both single-gene mutations we evaluated perturb the developing brain—*slc6a3* disrupting the physiology of dopamine cells (*26*) and *scn1lab* interfering with the specific tuning and connectivity of the neural circuits underlying the OMR (*28*)—in developing larval zebrafish. Both affect κ but *w* much less so. The effect on κ may explain why the mutations change the effective integration of noisy whole-field motion and, consequently, the ability to integrate motion stimuli. The negligible effect on *w* is explained by the fact that parental contributions are the same. Our approach of separating focus and competence allows for the isolation of behavioral phenotypes in larval zebrafish, which may be due to either inherited or environmental contributions, and can be subjected to detailed dissection at the circuit level in future experiments.

## MATERIALS AND METHODS

### Behavioral experiments

All experiments followed protocols approved by the Harvard Institutional Animal Care and Use Committee. Behavior experiments were carried out on zebrafish larvae at 7 days postfertilization. Zebrafish swim in a circular dish with a diameter of 12 cm made with a clear acrylic base of 0.125 in. (3.175 mm) in thickness and a black acrylic wall with a thickness of 0.25 in. (6.35 mm). At the start of a day’s experiment, 50 ml of filtered water from the fish facility is added to each dish to keep the initial water volume constant. Experiments are conducted between 9 a.m. and 5 p.m. Fish swimming is recorded from a camera placed above the dish. The camera is a Grasshopper3 from FLIR systems, model GS3-U3-41C6NIR attached to a Navitar Zoom 7000 lens and a Hoya R72 mm infrared filter. Stimuli are presented to the bottom of the dish using a projector (AAXA P300) and diffusive paper. Infrared illumination (Univivi infrared light-emitting diode lamps) is used to detect fish while ignoring stimuli that have components only in the visible light spectrum. Online tracking of zebrafish is done at 90 Hz through custom software written in Python (version 3.8) and C++ (integrated into Python through dynamic link libraries) to identify fish position, angle turned, and bouts (zebrafish swim in punctuated movements—these discrete events are identified as bouts and consist of a burst in tail movement, resulting in a change in orientation corresponding to the single “bout”). Both camera recording and stimulus presentation are aligned and synchronized for spatial correctness. Our setup allows us to record data from 16 fish (with independent dishes, camera, and stimulus projection) simultaneously. Structures to hold cameras and dishes in place and breadboards to house the entire setup were sourced exclusively from ThorLabs (optical rails, breadboards, connectors, and screws) and assembled in house. Four cameras are connected to a single computer, and each computer runs an Intel i9 processor, 64GB RAM, 4 TB hard disk, hi-speed USB data transfer card, NVIDIA GeForce GTX 1060 GPU.

A single experiment consists of 30 trials where each trial is a 180-s chunk with the first 150 s showing 0% coherence dot motion and the last 30 s showing the desired coherence level (typically 100%) with dots moving to the left or right. To keep the number of presentations to the left and right constant, we ensure that every successive pair of trials consists of one leftward stimulus and one rightward stimulus, chosen in random order. All initial experiments were carried out with a total of 64 fish. Perturbation experiments typically have 32 fish in each group (except mutant fish where numbers are dependent on genotyping done postexperiment).

### Visual stimulus

Randomly moving dots with a speed of 1.8 cm/s and a lifetime of 200 ms are projected onto a circle with a diameter of 12 cm. The stimulus consists of 1000 white dots placed on a dark background and is programmed on Python using panda3d and OpenGL Shading Language. At any point, the stimulus maintains a constant orientation with respect to the fish’s body axis. This is achieved by rotating the stimulus by the same angle turned by the fish in each bout. The stimulus presentation software and fish tracking software have a communication loop to facilitate this.

### Data analysis

The bout-wise information on fish position and orientation is used to determine the baseline bout rate, stimulus bout rate, angle turned, and performance. Bout rates are computed by dividing the number of bouts in the desired time window by the length of the window. We exclude durations where the fish was not tracked (identifiable through error codes from the online tracking software) and ignore bouts that are initiated at a radius greater than 90% of the total distance to the edge of the dish. This removes any forced turns arising from being at the wall and accounts for bout rate adjustment based on the time window in which the fish was detected. Given that the stimulus-induced turning may have residual effects on swimming in the baseline period for the successive trial, we ignore the first 30 s of every baseline period in our analysis. The angle turned in a bout is computed using the difference in fish orientation before a bout is initiated and after a bout is completed. Performance is computed on the basis of the number of turns made in the direction of the stimulus. We treat 0 as a 50% contribution to correctness. Anything greater is considered a rightward turn, and anything lesser is considered a leftward turn. For all turn angle distributions, we use a bin size of 5 between (−180,180). Performance scores are rescaled to be within a range of [−1.0,1.0] such that the negative scores correspond to turns preferentially against the direction of the stimulus and positive scores correspond to turns preferentially in the direction of the stimulus. Distributions of performance scores are calculated in bins of 0.1 on the performance score, and distributions on bout rate are computed in bins of 10 bouts/min. Streaks are defined as successive turns in the same direction. We compute how many turn angles have the same sign and treat that set as a single streak. The radius is computed on the basis of the fish position at the start of each bout. The tracking software rescales the circular dish to a unit circle; therefore, radius values will lie within [0,1]. Plots of performance against the average radius report values for radius binned in units of 0.1.

### Coin flip model

We model the probability of turning as a binomial process whereby the animal can turn left or right randomly (according to a coin flip) in every bout. The total number of bouts in each trial’s baseline period determines how many coin flips must be carried out. We average the probability of obtaining a certain performance given a certain number of coin flips and lastly average these scores across all observed coin flip sequences to generate a theoretical estimate of the performance score distribution (e.g., for a single coin flip, you can only obtain a performance score of +1 or −1, but a finer resolution is possible for a greater number of flips—here, flips are analogous to the number of bouts).

### Modeling performance distribution

We use a Gaussian distribution centered at the chance level (zero) and a gamma distribution normalized within the performance score range to fit the performance distributions. The motivation for the gamma function arises from the shape of the performance score distributions across parameter combinations in the DDM—they span both an exponential nature near high-performance scores and Gaussian nature in the same region, particularly in the absence of state transitions. The gamma function captures these extreme features under the same distribution. We rescale the performance scores to [0,1] just for fitting purposes. Once the scores are normalized to the range of [0,1] in bins of 0.5, we use the curve_fit subroutine from the scipy library to find suitable *w* and κ that satisfy

$$P=\left(1-w\right)\times {Z^{-1}}_{\text{Gauss}}\times N\left(0,0.18\right)+w\times {Z^{-1}}_{\text{Gamma}}\times \text{Gamma}\left(\frac{1}{\kappa },15\right)$$

$$\text{Gamma}\;\left(a,b\right)=x^{a-1}e^{-\mathrm{bx}}$$

where *P* is the final performance distribution. $Z_{\text{Gauss}}$ and $Z_{\text{Gamma}}$ are integrals over the Gaussian and gamma distributions, respectively, required to restrict the distribution between [0,1]. For visualization, we still report the distribution over performance scores of [−1,1], which is a simple rescaling of the [0,1] range. The values of 0.18 and 15 in the above equation are the standard deviation of the Gaussian function (σ) and the rate parameter of the gamma function (θ), respectively. σ is the standard deviation of a Gaussian distribution that equals the baseline performance score distribution (fig. S1D). This is a consequence of framing this model as combining a baseline (disengaged) response profile with an engaged response profile. θ is determined by matching subsequent fits constrained by the value of σ = 0.18 and by comparing the distribution of performance scores during stimulus, yielding θ = 15. Like other parameter tunings, we again perform least-squares minimization within a suitable range of θ. We generate 5000 bootstrapped samples from the target distribution of scores and fit each set of samples to get a confidence estimate for our parameter estimates *w* and κ. The error bars on parameters only denote this estimation accuracy through bootstrapping and do not imply anything about statistical significance across values. Every fit has a randomized initial condition to ensure that the solution space is sufficiently explored and that the fitting procedure is not stuck in a local minimum by chance.

### Fit evaluation

Fits of the performance score distribution using the Gaussian-gamma model were evaluated using mean squared error (MSE) from the experimentally observed distribution. As a baseline, we also evaluate the MSE of a null model from the experimental distribution to highlight the accuracy of the fitting procedure and provide context for the error values. As a null model, we choose a uniform distribution over performance score bins. Given that we use 21 bins to calculate performance score distributions, the null model has a probability of 1/21 for each bin, and the MSE between this model and the experimentally observed distribution is computed. Furthermore, we report the ratio between the fit MSE and the null MSE, expressed as a percentage, to quantify the improvement using our Gaussian-gamma fit approach.

### Statistical tests for *w* and κ

Given that our claims involve the relative influence of parental origin/raising environment/acute perturbations on *w* or κ, we perform a statistical test to determine whether the change in parameter value before and after the perturbation is significant. This is performed as a permutation test using the following procedure: We pool the data from the two groups of fish being tested and randomly repartition the fish into two new groups. We estimate *w* and κ for each of these repartitioned groups and compute the difference between the corresponding parameter values from each group (κ_1_ − κ_2_ and *w*_1_ − *w*_2_). This partitioning and difference estimation is repeated 10,000 times to obtain a distribution of differences for *w* and another for κ. Last, we calculate the probability that our observed differences in *w* and κ values from the original groups being tested have a value that is at the extremes of the computed difference distributions. This provides a *P*_bootstrap_ value that is used as a measure of statistical significance for all parameter comparisons between κ and *w*.

### HMM of behavioral performance

We fit an HMM of behavioral performance. The gamma HMM describes a time-varying process where the observed data *x_t_* is a sample from a gamma distribution with parameters associated with one of *k* unobserved latent states *z_t_* = *k*, *p*(*x_t_*|*z_t_* = *k*) ∼ Gamma(α*_k_*, β*_k_*). The initial latent state *z*_0_ is drawn from a probability distribution π and changes according to a Markov process with a transition matrix *A* such that on a given trial, the probability of transitioning from the current state *i* to another state *j* is *p*(*z*_*t*+1_ = *j*|*z_t_* = *i*) = *A_ij_*. The set of free parameters for the gamma HMM Θ ≡ π, *A*, α, and β is fit to the observed optomotor performance data using expectation maximization. We fit HMMs with 1 to 10 discrete states using fivefold cross-validation and evaluated the likelihood on held-out test data. We find that a two-state model considerably outperforms a single-state model (equivalent to a gamma random variable) and that adding additional hidden states does not meaningfully increase the validation set log likelihood (Fig. 2D, right). Therefore, we choose a two-state model for subsequent analyses. The resulting model parameters have two states that correspond to an “engaged” state, where the mean performance is high and the standard deviation is low, and a “disengaged” state, where the mean performance is low and the standard deviation is relatively high. The states have duration distributions with a median of five trials but a long tail with many states lasting over 10 trials (Fig. 2D, left).

### Fish care

Zebrafish are housed in the fish facility of the Sherman Fairchild building and Biological Laboratories buildings. The temperature of the facility is maintained at 27°C. Adult zebrafish from the AB WT strain are crossed pairwise in the afternoons, and embryos are collected in the morning on the following day. This is counted as day 0 or the day of fertilization. All experiments are done 7 days postfertilization. Procedures are in accordance with the guidelines set by the Institutional Animal Care and Use Committee in the Biological Laboratories and Sherman Fairchild facilities. Fish embryos are raised in the water circulating in the fish facility after filtering using a 30-μm filter to remove any unintended impurities present in the water. The same water is used to conduct experiments as well. The larval zebrafish are fed on day 5 and day 6 postfertilization but not on the day of the experiment (day 7). The Biological Laboratories facility uses paramecia and Sherman Fairchild uses rotifers to feed the larvae. No more than 100 embryos are placed in a single large petri dish (diameter of 15 cm) during raising. Before the experiment, fish are always placed on a white paper towel and allowed to rest for 10 min before proceeding with the experiment. This ensures that all zebrafish have similar illumination beneath them before being placed in the experiment chamber.

### Mutant line genotyping

To identify the genotype of mutant fish postexperiment, we suspend larval zebrafish in 25 μl of an alkaline lysis buffer solution (0.2 mM EDTA and 25 mM NaOH). This tissue is heated at 95°C for 1 hour. These samples are held at 4°C for up to 24 hours, after which 25 μl of neutralizing buffer solution (40 mM tris-HCl) is added. After vortexing at 100 rpm for 2 min, samples, hereon referred to as gDNA, are stored at 4°C for high-resolution melt curve analysis (HRM). The reference curve-based HRM protocol requires preparation of 10-μl sample reactions, with 1.0 μl of isolated gDNA, 5.0 μl of MeltDoctor HRM Master Mix, 0.6 μl of primers for the scn1lab and slc6a3 lines, and 2.8 μl of nuclease-free water. Previously isolated WT, heterozygous, and homozygous gDNAs are used as controls. We run triplicates for all samples including controls in a 384-well plate using the QuantStudio 6 Flex System. The program consists of a 10-min hold at 95°C, followed by 40 cycles of 0.15 s at 95°C and 1 min at 60°C. The last step is a continuous HRM of 10 s at 95°C, 1 min at 60°C, 15 s at 95°C (dissociation stage), and 15 s at 60°C. After completion, the aligned melting curves are compared with controls and assigned to the corresponding genotype.
