# Supplementary material for: Attentional switching in larval zebrafish
Source: Sci Adv. 2025 Oct 3;11(40):eads4994. doi: 10.1126/sciadv.ads4994 (PMC12494005; doi:10.1126/sciadv.ads4994)
Supplement: Supplementary file 1 — Supplementary Text S1 Figs. S1 and S2 [file sciadv.ads4994_sm.pdf]

Science Advances
AAAS

Supplementary Materials for

Attentional switching in larval zebrafish

Kumaresh Krishnan *et al.*

Corresponding author: Kumaresh Krishnan, kumaresh_krishnan@fas.harvard.edu

Sci. Adv. **11**, eads4994 (2025)
DOI: 10.1126/sciadv.ads4994

This PDF file includes:

Supplementary Text S1
Figs. S1 and S2

SUPPLEMENTAL TEXT 1

1.1 Drift Diffusion Model

In order to study putative underlying algorithmic processes for these behavioral phenomena, we adapt a previously implemented modeling approach, in which the behavior of a larval zebrafish in the context of the coherent dot motion paradigm can be captured by the drift-diffusion to bound (DDM) model. This model has been successfully applied in comparable assays in humans, non-human primates and rodents (fig. S2F).

In the classical DDM, sensory information is integrated over time as a decision variable *X(t)*. When *X(t)* crosses a certain threshold *T*, the organism reports a 'decision'. This reflects a transition from passive observation below threshold to full activity above threshold. Here we use the 'swim direction' in each bout as a readout for decision making. Since fish constantly swim, our adaptation of the DDM has a non-zero rate of 'decisions' (termed r_B) below threshold, and the decision triggers a transition to a higher reporting rate above threshold (termed r_A). The direction of a swim is based on the sign of *X(t)* - rightward for positive values and leftward for negative ones. In this context we can think of the fish as compulsively reporting the value of *X(t)* at every executed bout that occurs roughly once a second, and where the outcome of each report depends only on the sign of *X(t)*. The role of the threshold *T* is then reduced to simply increasing the 'reporting rate' from r_B to r_A.

The core of our implementation of the DDM is a leaky integrator (see A2) and an interpretation for its parameters are as follows: (i) *C(t)* is the level of motion coherence in the dots, ranging from 0 for no coherence to 1 for fully coherent motion - a non zero *C(t)* generates the 'drift' in the model, (ii) τ is the time constant that sets the temporal window for integration of motion information, whereby high τ implies slow leak and long 'memory', (iii) noise in the input signal $\eta(t)$ is parameterized by its standard deviation Ω, (iv) T is a threshold on the integrated motion information above which bout rate simply switches to higher values.

Since motion coherence information *C(t)* is zero during the baseline period this implies that r_B reflects the baseline bout rate as the integrated information *X(t)* is largely below threshold. During the stimulus period, however, *X(t)* can spend varying proportions of time above and below threshold depending on the values of the model parameters τ, Ω and *T*. Consequently, the observed stimulus bout rate is a mixture of the reporting rates r_A and r_B (see fig. S2G for an example). In its most recent implementation in larval zebrafish, the DDM uses two fixed bout rates, one below the threshold (r_B=0.6s^{-1}) and one above (r_A=2.3s^{-1}) for all simulations(*11*). Since zebrafish exhibit a distribution of bout rates, both in baseline and stimulus periods (Fig. 1D), we investigate how performance is impacted over a range of r_B and r_A values by simulating the model for the same number of repetitions as in the experiment i.e. 64 fish and 30 trials. For each trial, a new r_B and r_A are chosen (see A2).

As a first control, we compute the psychometric curve in the modeled experiment (fig. S2I), and find that it qualitatively matches the experimental results. The DDM takes coherence strength as an input with values between 0 and 1.0. The lack of non-linearity applied to this input is likely to explain the slight differences between fig. S2I and Fig. 1C causing saturation of performance

scores for coherence strengths > 0.5. We confirm that the performance increases with longer wait times for the first bout after stimulus onset (fig. S2J). Simultaneously optimizing the model parameters requires solving a multi-objective optimization problem which is analytically infeasible for this problem. In line with the optimal values obtained in earlier work(*11*), we chose values of τ=2.0 s, *Ω*=0.15, T=1.0 for the model parameters (see A2). The value for *Ω* differs from earlier work (*Ω*=0.2), since we are not using a constant r_B and r_A across all simulations.

We next wanted to know whether the performance of each animal depends on its bout rate, either during the stimulus or during the preceding baseline period. We find, in both the experiment and model, a negative correlation of performance with the baseline bout rate (fig. S2K top, bottom), indicating that fish which swim more slowly under baseline conditions display higher performance scores during the subsequent stimulus period. Performance in this case is evaluated in the stimulus period that immediately follows the corresponding baseline period. Since the baseline bout rate is representative of r_B, we attribute such an increase in performance to a lower ratio of r_B/r_A , where the fish is making relatively fewer bouts below the threshold. The region below threshold presents a higher probability for the decision variable *X(t)* to oscillate between positive and negative values, increasing the chance of making mistakes when r_B is high. In contrast, the observed stimulus bout rate shows no strong effect on performance in neither the experiment nor the model (fig. S2L top, bottom). Together, these results show that the drift-diffusion model can recreate the trends in average performance across bout rate variation. Further, the baseline bout rate and stimulus bout rate are strongly correlated (fig. S2M) which indicate that periods of high baseline swimming are followed by periods of high stimulus evoked swimming. We next proceed to look at the structure across individual trials rather than averages, to determine whether the model captures this low-level structure as well.

Using this more detailed analysis we find several discrepancies between model and experiment. First, the data show a drop in performance in later trials (fig. S2N black line), which was not captured by a model implementing only the DDM (fig. S2R). In principle, this decrease in performance could be explained within the framework of the model if baseline bout rate increases over consecutive trials. Indeed, we find that baseline bout rates marginally increase over the course of the experiment (fig. S2S black line), which might account for the decrease in performance. This increase also demonstrates that fish are not simply getting 'tired' over the 90-minute time course that an experiment lasts.

However, we find that modeling consecutive trials with incrementally increasing r_B fails to capture the dramatic drop in performance we observe in the experiments (fig. S2N). Second, we observe that performance scores in the experiment span the full range between -1 and 1, while the model generally predicts scores between 0 and 1 (fig. S2E). This lack of scores close to -1 in the model is explained by the fact that consistently negative values of the integrator variable in the presence of motion evidence are highly improbable. Therefore, this implementation of the drift-diffusion model cannot capture the structure across trials in the experiment and is likely an incomplete description of decision making in larval zebrafish.

We emphasize that the relatively large spread of scores between -1 and 1 in both model and experiment (fig. S2T) is achieved through the memory component in larval zebrafish swimming, where zebrafish make multiple consecutive bouts in the same direction (see Fig. 1H).

1.2 Implementation

The drift diffusion model operates on the following equation governing how a motion signal is integrated over time:

$$\tau \frac{dX}{dt} = -X(t) + C(t) + \eta(t)$$

$$\eta(t) \in N(0, \Omega)$$

where *X(t)* is the integrated information, *C(t)* is the coherence level (value of 0 for no coherence and 1 for 100% coherence), Ω is the standard deviation of the noise and τ is the time constant of integration (a measure of leakiness). We simulate trajectories i.e. *X(t)* for the entire experiment duration, using the above equation for the same number of repetitions as the experiment (64 fish, 30 trials so 1920 repetitions). The time step for integration is $\Delta t = 10ms$. Bouts are samples from this trajectory at a rate that depends on the threshold T. Below this threshold, bouts are generated at rate r_B and above this threshold, bouts are generated with a rate r_A. The sign of *X(t)* determines the distribution of turn angles that the simulated fish draws from for a bout. Using r_B and r_A, we first determine whether a bout will be generated in the given time step from *X(t)*. We then pick the direction of the bout to be rightward if *X(t)* is positive, otherwise leftward. The specific turn angle of the bout is picked randomly drawn from the empirical distribution of turn angles for a given direction as shown in Fig. 1B. We constrain the interbout interval to be at least 200ms, based on empirical evidence (If the integration process indicates that a bout must be generated in a timestep that is less than 200ms after the previous bout, we do not generate a bout and move to the next time step).

To present a one-one comparison between experiment and model, we choose r_B and r_A in the following manner. Each trial in the experiment has an associated baseline bout rate. Since this is reflective of r_B, every trial in the model simulation uses the same value of r_B as the baseline bout rate from the corresponding trial in the experiment. The observed stimulus bout rate in the experiment is a mixture of r_B and r_A. Therefore, we choose r_A to match the stimulus bout rate from the corresponding trial in the experiment. This value is increased by 20% to negate the contribution of r_B to the stimulus bout rate, and recover an approximation for r_A. In this manner, each of the 1920 trials in the experiment are appropriately matched by the trials in the simulation.

The tuning of model parameters is done by minimizing the mean squared error between performance score variation with bout rate in the experiment and the model (fig S2K,L top). These values are not rigorously tuned since these minor variations are irrelevant to the larger point of the original drift-diffusion model not capturing the structure across trials.

All data from the simulation is stored in the same format as the experiment to allow the same analysis scripts to run on both data.

1.3 DDM With Attentional Switching

Given the two observed components in the performance distribution scores (Gaussian and Gamma), we sought to extend the drift-diffusion model through a simple two state transition between a disengaged state and an engaged state, where the transition probabilities to stay in the engaged and disengaged states are dictated by *p* and *q* respectively (Fig. 2B right). We rely on two

key observations to determine how the value for p and q are modulated in the model: (i) p is high when r_B is low, since r_B is representative of the baseline bout rate and we know performance is higher at low baseline bout rates. (ii) p decreases in later trials, since we observe that average performance drops with time in the experiment.

The transition to the disengaged (with respect to the optomotor response) state is implemented simply by removing the input motion evidence signal, i.e., by setting *C(t)* to zero. This is equivalent to drawing the drift rate from a bimodal distribution (0 or 1), rather than keeping it constant across trials (or drawing from a normal distribution), as is common in standard implementations of the DDM.

Essentially, under the updated framework, zebrafish follow the DDM strategy while in an engaged state and ignore motion information while in a disengaged state - identical to the baseline period behavior in the DDM (purely a diffusion process since there is no 'drift' caused by *C(t)*). The new model parameters of τ=2.8s, Ω=0.08, T=0.8 reflect these two states and match the spread of performance scores in both baseline and stimulus periods remarkably well (see A4 for parameter tuning details).

Notably, the drop in performance with increasing r_B is now achieved by (i) an enhanced probability to switch into the disengaged state at higher r_B values as p is negatively correlated with r_B (fig. S2O recovers the missing trend from fig. S2R and S2N) (ii) previously explained phenomenon of r_B causing more mistakes while in the engaged state (fig. S2O). Since p is computed trial by trial, the average performance drop observed during later trials in the experiment is now captured in the model as well (fig. S2O).

1.4 Implementation of DDM With Attentional Switching

We incorporate a two-state transition governed by parameters p and q denoting the probability of staying in an engaged state and disengaged state respectively. The base values for these parameters p and q are obtained from the transition matrix of the HMM model (Fig. 2B right). These values are modulated by the baseline bout rate r_B and trial number as a proxy for time in the experiment. We quadratically map r_B and trial number (t_N) to the range [0.5,2] where the end points indicate halving and doubling the base probability. Next, we compute a weighted average of r_B and t_N as follows $p_{scale} = 0.75 * r_B{}^{norm} + 0.25 * t_{norm}$. The scaling of p and q follows from our empirical observations on the variation of performance with r_B and t_N. Quantitatively, we scale p by $1/p_{scale}$ and q by p_{scale} Once the state is determined at the start of each trial, we turn off the stimulus (set *C(t)* to 0) if the model picks an inattentive state. Parameters are fine tuned to values of τ=2.8s, Ω=0.08, T=0.8. We tune τ by evaluating the baseline distribution of performance scores generated for various values of τ. The final value is chosen as the τ corresponding to the width of the Gaussian (σ) that minimizes mean squared error with the *baseline distribution* of scores (fig. S2P, S1D), i.e. σ=0.18. Ω and T do not depend on σ since they are irrelevant to the baseline period (fig. S2Q). Therefore, the values for Ω and T are tuned by minimizing mean squared error with the distribution of performance scores *during stimulus*, over a search of the parameter space.

SUPPLEMENTARY FIGURES

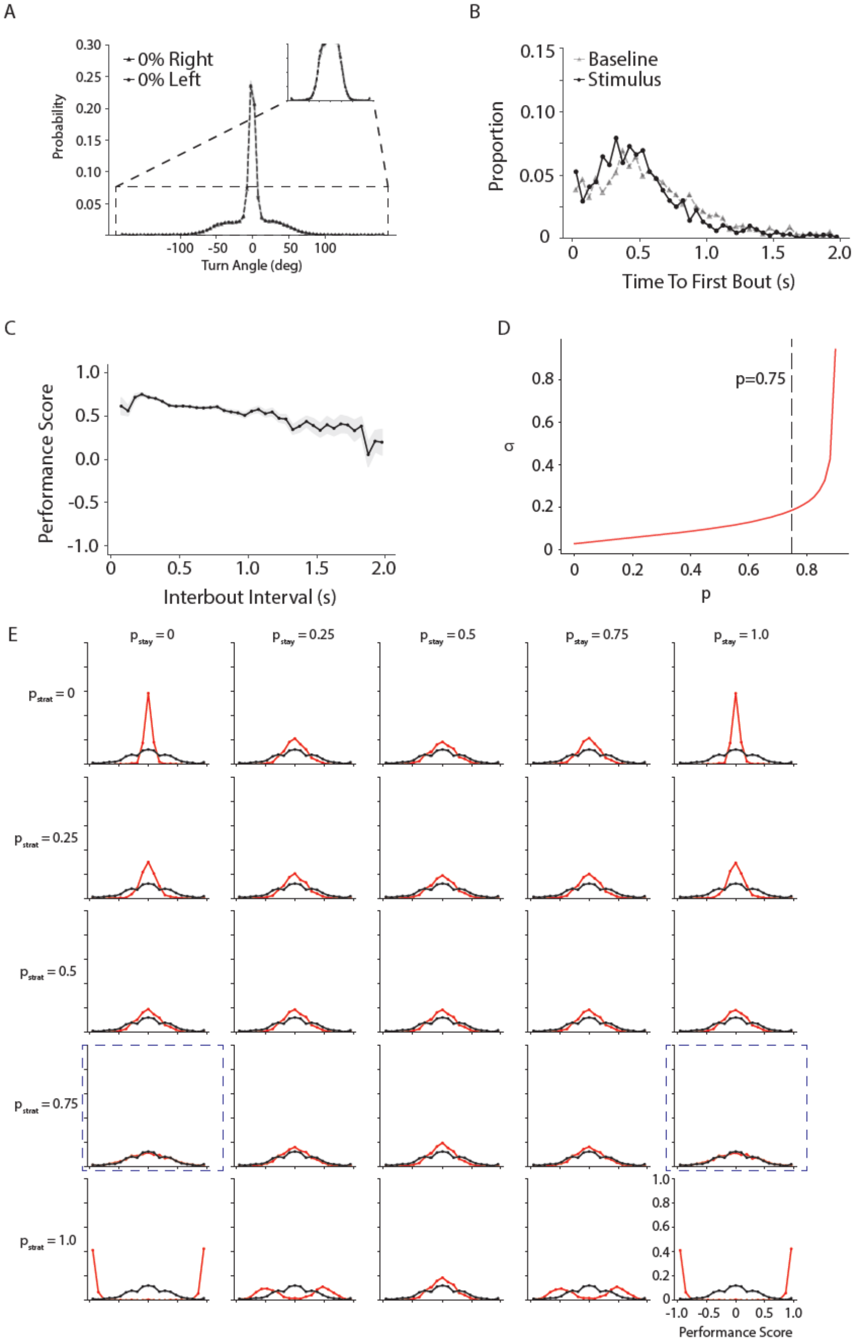

Figure S1. (A) Turn angle distributions for 0% coherence leftward and rightward in circles and triangles respectively – responses are symmetric as evident in inset (B) Proportion of all first bouts after stimulus onset with a specific wait time – large wait times are rare events (C) Average performance score for all bouts other than the first, binned by interbout interval – waiting longer during stimulus period does not improve performance (D) Bias required in the coin flip model to match experimentally observed performance score distribution ‘p’ plotted against the width of a Gaussian function that fits this distribution of scores ‘σ’ (E) Different parameter combinations of a two state model (left bouts and right bouts) where probability to stay in the same state over successive bouts increases from left to right and probability to pick bouts from the wrong direction in a given state increases from top to down – candidate values are shown in dashed boxes

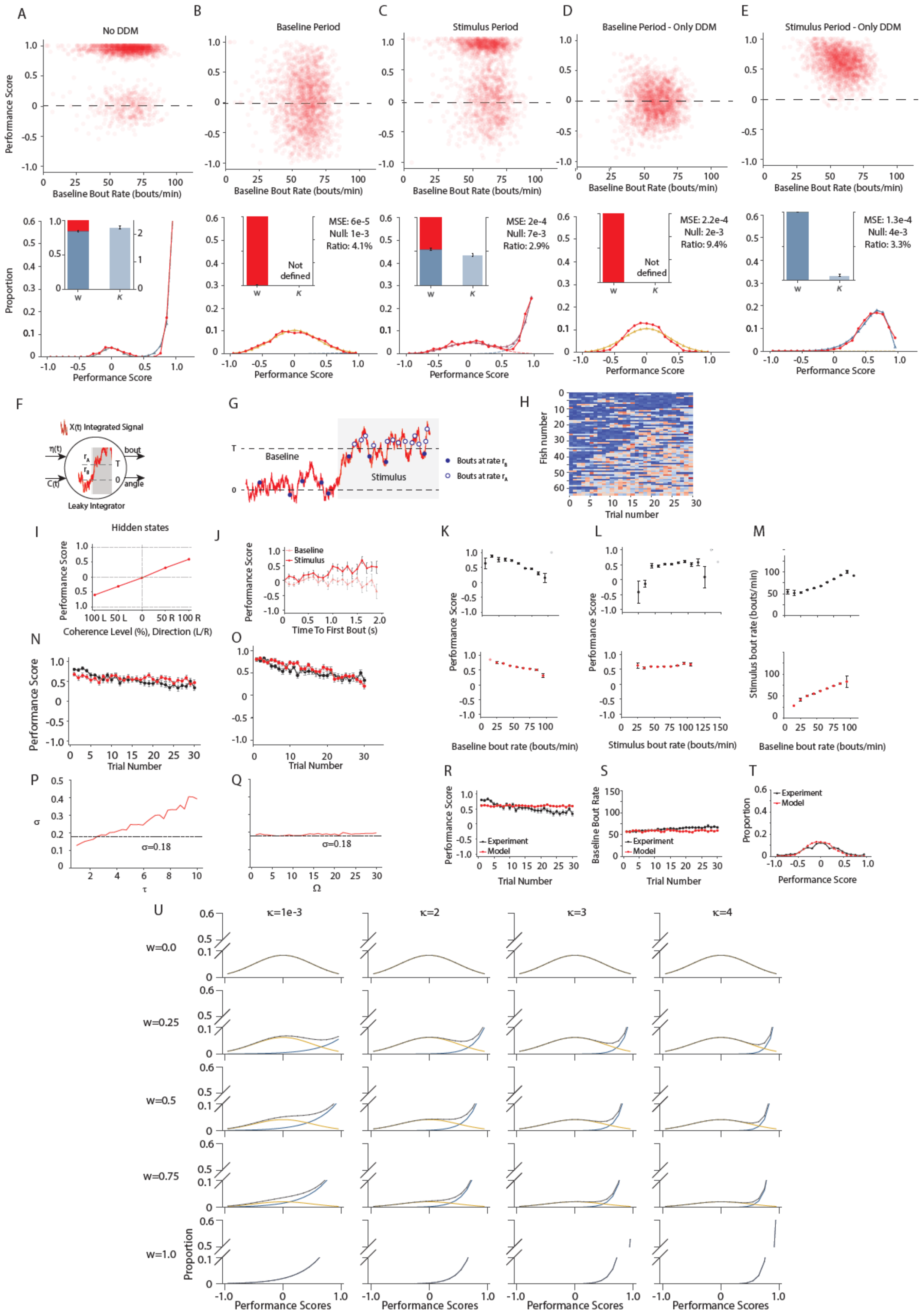
A
No DDM
Performance Score
Baseline Bout Rate (bouts/min)
B
Baseline Period
C
Stimulus Period
D
Baseline Period - Only DDM
E
Stimulus Period - Only DDM
Proportion
Performance Score
w
κ
Not defined
MSE: 6e-5
Null: 1e-3
Ratio: 4.1%
MSE: 2e-4
Null: 7e-3
Ratio: 2.9%
MSE: 2.2e-4
Null: 2e-3
Ratio: 9.4%
MSE: 1.3e-4
Null: 4e-3
Ratio: 3.3%
F
X(t) Integrated Signal
η(t)
C(t)
bout
angle
T
0
Leaky Integrator
G
T
0
Baseline
Stimulus
Bouts at rate r_B
Bouts at rate r_A
H
Fish number
Trial number
I
Hidden states
Performance Score
Coherence Level (%), Direction (L/R)
J
Baseline
Stimulus
Time To First Bout (s)
K
L
M
Performance Score
Baseline bout rate (bouts/min)
Stimulus bout rate (bouts/min)
Stimulus bout rate (bouts/min)
N
O
Performance Score
Trial Number
P
Q
σ
σ=0.18
τ
Ω
R
S
T
Experiment
Model
Performance Score
Baseline Bout Rate
Trial Number
Proportion
Performance Score
U
κ=1e-3
κ=2
κ=3
κ=4
w=0.0
w=0.25
w=0.5
w=0.75
w=1.0
Proportion
Performance Scores

Figure S2. (A) Point cloud of 1920 baseline bout rate, performance pair simulated by sampling from the attentional switching state transition (B) Point cloud of 1920 baseline bout rate, performance pair simulated for the baseline period by combining attentional switching with DDM (C) Point cloud of 1920 baseline bout rate, performance pair simulated for the stimulus period by combining attentional switching with DDM (D) Point cloud of 1920 baseline bout rate, performance pair simulated for the baseline period using only DDM (E) Point cloud of 1920 baseline bout rate, performance pair simulated for the baseline period using only DDM (F) Schematic of the drift-diffusion model for decision making (G) Sample trajectory for *X(t)* in red showing bouts generated below threshold at rate r_B in shaded blue circles and a faster rate r_A above threshold shown in open blue circles - baseline bout rate is r_B here, stimulus bout rate is mixture of r_A and r_A (H) Heatmap showing performance score for each fish across trials using attentional switching and DDM (I) Psychometric curve for average performance at each coherence level generated by the model - for visualization purposes, only the 0 (chance) to 1.0 (fully correct) region of scores are shown on the y-axis (J) Variation of performance with time taken for the first bout after stimulus onset – light red triangles show baseline period and red circles show stimulus period, averaged in 100ms bins (K) Variation of performance (during stimulus period) with bout rate in baseline period, averaged in 10 bouts/min bins; top – Experiment, bottom – Simulation (L) Variation of performance (during stimulus period) with bout rate in stimulus period, averaged in 10 bouts/min bins; top – Experiment, bottom – Simulation (M) Variation of stimulus bout rate with baseline bout rate, averaged in 10 bouts/min bins; top - Experiment, bottom - Simulation (N) Average performance score across all fish over trial number in experiment (black) and model (red) with transition rate parameter p depending only on r_B and not trial number (O) Average performance score across all fish over trial number in experiment (black) and simulation (red) with transition rate parameter p depending only both r_B and trial number (P) Variation of parameter τ with the standard deviation of the Gaussian function associated with inattentive state 'σ' – dashed line at $\sigma = 0.18$ matching the best fit to experiment, corresponding with τ=2.8s (Q) Width of inattentive Gaussian (baseline score distribution) is independent of noise term Ω in the drift diffusion process (R) Average performance score across all fish over trial number in experiment (black) and simulation (red) using only DDM without any state transition (S) Average baseline bout rate across all fish over trial number in experiment (black) and simulation (red) using only DDM without any state transition (T) Distribution of performance scores in experiment (black) and simulation (red) combining attentional switching with DDM (U) Sample fits for different combinations of w and κ – circles show combined distribution, golden shows Gaussian component and blue shows Gamma component
